# Supplementary material for: Peer-led sexual and reproductive health education and risky sexual behavior among adolescent girls and young women in rural Uganda: A quasi-experimental pre-post study
Source: PLOS Glob Public Health. 2026 Sep 28;6(9):e0006165. doi: 10.1371/journal.pgph.0006165 (PMC13618902; doi:10.1371/journal.pgph.0006165)
Supplement: Supplementary file 1 [file pgph.0006165.s002.docx]

# Questionnaire (Researcher administered) for Baseline data

| **Study Title** | Contextual factors and Effectiveness of Community-Based Peer-Led Sexual and Reproductive Health Education on Risky Sexual Behaviour for adolescent girls and young women in Moroto District, Karamoja Region. |
| --- | --- |
| **Short title** | Community-based Peer-led sexual and reproductive health education study |
| **Objectives 2-4** | 2. To determine the effect of peer-led community-based SRH education on risky sexual behavior. 3. To establish the effect of the peer-led community-based sexual reproductive health education on SRH knowledge. |

| **Unique Identification Number:** | | **Village Code:** | **Date:** |
| --- | --- | --- | --- |
| **S/N** | **QUESTION** | **RESPONSE** | **SKIP** |
|  | **Background characteristics** |  |  |
| 1. | Name of the Village | ___________________ |  |
| 2. | How old are you? | ___________________ |  |
| 3. | What is your religion? | 1. Christian 2. Muslim 3. Others (specify) _____ |  |
| 4. | Marital status | 1. Single 2. Married/Cohabiting 3. Separated/divorced 4. Widow |  |
| 5 | Age at marriage | ____________ | If not married skip to Qn 6 |
| 6 | Employment status | 1. Employed 2. Not employed | If not  employed, skip to question 8) |
| 7 | Nature of employment | 1. Vendor/hawker 2. Shop keeper |  |

|  |  | 3. Maintenance/support 4. Security 5. Teacher 6. Health worker 7. Farmer 8. Others (specify) |  |
| --- | --- | --- | --- |
| 8 | Are you currently in school? | 1. Yes 2. No |  |
| 9. | What is your highest level of education? | 1. None 2. Primary 3. Secondary 4. Tertiary/ institution 5. University |  |
| 10. | What is the status of the parents | 1. Both father and mother are alive 2. Only father alive 3. Only mother alive 4. Both Father and mother dead |  |
| 11. | Who do you live with? | 1. Both father and mother 2. Only father 3. Only mother 4. Spouse 5. Relatives 6. Friends 7. In-laws 8. Others (Specify) |  |
| 12 | Nature of parent/caretaker’s employment | 1. Vendor/hawker 2. Shopkeeper   3.  Maintenance/support   4. Security 5. Teacher 6. Health worker 7. Farmer 8. Others (specify) |  |
|  | **SRH Indicators** |  |  |
| 13 | Have you ever had sexual intercourse | 1. Yes 2. No | *If no, skip to question 19* |
| 14 | If yes, at what age was your first sexual encounter? | _______________ |  |

| 15 | Do you have any biological children? | 1. Yes 2. No | *If no, skip to question 19* |
| --- | --- | --- | --- |
| 16 | If yes, how many children do you have? | ______________ |  |
| 17 | At what age did you have your first child? | ______________ |  |
| 18 | Was the recent pregnancy planned? | 1. Planned 2. Not planned |  |
| 19 | How many HPV doses did you receive before the age of 15 years | 1. Yes 2. No |  |
| 20 | Is there any time, when you wanted modern contraceptives and they weren’t available? | 1. Yes 2. No |  |
| 21 | Have you been diagnosed with STI in the last 6 months? | 1. Yes 2. No |  |
| **Risky Sexual behavior** | | |  |
| 22 | Have you had sexual intercourse with a commercial sex worker in the past 3 months? | 1. Yes 2. No |  |
| 23 | Have you had sexual intercourse with multiple sexual partners? | 1. Yes 2. No |  |
| 24 | In the past 3 months have you had sex without a condom? | 1. Yes 2. No |  |
| 25 | Have you had sexual intercourse under the influence of substance use? | 1. Yes 2. No |  |
| 26 | **Summary of risky sexual behavior**  Risky sexual behavior among AGYW (*Participants who report engaging in any one of the four behaviors will be considered to have engaged in risky sexual behavior*). | 1. Yes 2. No |  |
|  | **Knowledge on SRH** |  |  |
| 34 | Do you know any modern contraceptive methods? | 1. Yes 2. No |  |
| 35 | If yes, list them | ______________________ |  |
| 36 | Have you ever heard about any infections that people can get from sexual intercourse | 1. Yes 2. No |  |
|  | **Comprehensive HIV/AIDS Knowledge** |  |  |

| 27 | Do you know about HIV/AIDS | 1. Yes 2. No | If no skip to  QN 43 |
| --- | --- | --- | --- |
| 38 | How is HIV/AIDS transmitted | __________________ |  |
|  | **HIV/AIDS Misconceptions** |  |  |
| 39 | A healthy-looking person cannot have  HIV | 1. Yes 2. No |  |
| 40 | HIV can be transmitted by mosquito bites | 1. Yes 2. No |  |
| 41 | HIV can be transmitted by supernatural means | 1. Yes 2. No |  |
| 42 | A person can become infected by  sharing food with a person who has HIV | 1. Yes 2. No |  |
|  | **Self-reported perceived being infected with STIs including HIV** |  |  |
| 43 | It is possible to get an STI/HIV if you  have had unprotected sexual intercourse in the past 6 months | 1. Yes 2. No |  |
|  | **Self-reported perceived risk of becoming pregnant** |  |  |
| 44 | It is possible to get pregnant with one act of sexual intercourse without contraception. | 1. Yes 2. No |  |
| 45 | **Summary of knowledge on SRH**  (*Participants who report awareness of at least one modern contraceptive method, and ever heard about any infections that people can get from sexual intercourse and are aware of HIV, its transmission, and have correctly rejected two out of four common misconceptions about HIV/AIDS and report* the *perceived*  *risk of being infected with STIs including HIV and perceived risk of getting pregnant will be considered to have SRH knowledge).* | 1. Yes 2. No |  |
|  |  |  |  |

**Thank you for your participation**

# Questionnaire (Researcher administered) for End line data

| **Study Title** | Contextual factors and Effectiveness of a Community-Based Peer-Led Sexual and Reproductive Health Education on Risky Sexual Behaviour for adolescent girls and young women in Moroto District, Karamoja Region. |
| --- | --- |
| **Short title** | Community-based Peer-led sexual and reproductive health education study |
| **Objectives 2-3** | 2. To determine the effect of Community-based Peer-led sexual and reproductive health education on risky sexual behavior. 3. To establish the effect of the Community-based Peer-led sexual and reproductive health education on SRH knowledge. |

| **Unique Identification Number:** | | **Village Code:** | **Date:** |
| --- | --- | --- | --- |
| **S/N** | **QUESTION** | **RESPONSE** | **SKIP** |
| **Risky Sexual behavior** | |  |  |
| 1 | Have you had sexual intercourse with a commercial sex worker in the past 3 months? | 1. Yes 2. No |  |
| 2 | Have you had sexual intercourse with multiple sexual partners? | 1. Yes 2. No |  |
| 3 | In the past 3 months have you had sex without a condom? | 1. Yes 2. No |  |
| 4 | Have you had sexual intercourse under the influence of substance use? | 1. Yes 2. No |  |
| 5 | **Summary of risky sexual behavior**  Risky sexual behavior among AGYW (*Participants who report engaging in any one of the four behaviors will be* | 1. Yes 2. No |  |

|  | *considered to have engaged in risky sexual behavior*). |  |  |
| --- | --- | --- | --- |
| **Knowledge on SRH** | | | |
| 13 | Do you know any modern contraceptive methods? | 1. Yes 2. No |  |
| 14 | If yes, list them | _______________________  __ |  |
| 15 | Does consistent use of condoms during sexual intercourse reduce the risk of contracting HIV/AIDS? | 1. Yes 2. No |  |
| 16 | Can one having sexual intercourse with one faithful uninfected HIV partner reduce the risk of HIV/AIDS? | 1. Yes 2. No |  |
| 18 | A healthy-looking person cannot have  HIV | 1. Yes 2. No |  |
| 19 | HIV can be transmitted by mosquito bites | 1. Yes 2. No |  |
| 21 | A person can become infected by  sharing food with a person who has HIV | 1. Yes 2. No |  |
| 22 | It is possible to get an STI/HIV if you have had unprotected sexual intercourse in the past 6 months | 1. Yes  2.No |  |
| 23 | It is possible to get pregnant with one act of sexual intercourse without contraception. | 1. Yes 2. No |  |
| 24 | **Summary of knowledge on SRH**  [If yes in 13 AND yes in 15 AND yes in  16 AND yes in 17 and has comprehensive HIV knowledge AND yes in 22 and 23]. | 1. Yes 2. No |  |

**Thank you for your participation**
